# Post-splenectomy accessory spleen hyperfunction in children with hereditary spherocytosis: a rare case report and literature review

**DOI:** 10.3389/fped.2025.1572397

**Published:** 2025-05-26

**Authors:** Yuan-fei He, Shi-qin Qi, Jian Bian, Cheng-xiao Zhou, Pei Zhang

**Affiliations:** Department of Pediatric Surgery, Anhui Provincal Children’s Hospital, He Fei, Anhui, China

**Keywords:** hereditary spherocytosis, splenectomy, accessory spleen, children hereditary spherocytosis, children

## Abstract

**Objective:**

To enhance the understanding of splenectomy in children with hereditary spherocytosis, specifically focusing on the preservation of accessory spleens or partial splenectomy.

**Methods:**

A retrospective review of clinical data and surgical methods of a child with hereditary spherocytosis who underwent surgery for accessory spleen hyperfunction 7 years after splenectomy at the General Surgery Department of Anhui Provincial Children's Hospital, along with a literature review.

**Results:**

The child successfully underwent single-port plus one laparoscopic accessory spleenectomy. The surgery lasted 195 min, with an estimated blood loss of 600 ml. The postoperative hospital stay was 8 days, and at 6 months of follow-up, there were no complications such as bleeding, wound infection, thrombosis, or adhesive intestinal obstruction.

**Conclusion:**

For children with hereditary spherocytosis, the decision to preserve the spleen or accessory spleens during surgical treatment offers important reference value.

## 1 Introduction

Hereditary spherocytosis (HS) is a congenital hemolytic disorder characterized by defects in the red blood cell membrane, leading to increased susceptibility to hemolysis (1). Splenectomy is typically the definitive treatment for complications of HS, such as hemolytic anemia, jaundice, and splenomegaly. To prevent adverse outcomes, such as post-splenectomy infection, some researchers recommend partial splenectomy or preservation of accessory spleens in children with HS under 6 years old. This report presents a case of a 6-year-old child with HS who developed hyperfunction of the accessory spleen 7 years after undergoing splenectomy with preservation of the accessory spleen.

All procedures involving human participants in this study were conducted in accordance with the ethical standards of the institution and/or national research committees, as well as the 1964 Declaration of Helsinki and its subsequent amendments, or comparable ethical standards. Written informed consent for the publication of all materials showing patient details was obtained from the patient.

## 2 Materials and methods

### 2.1 Clinical data

**Figure 1:**
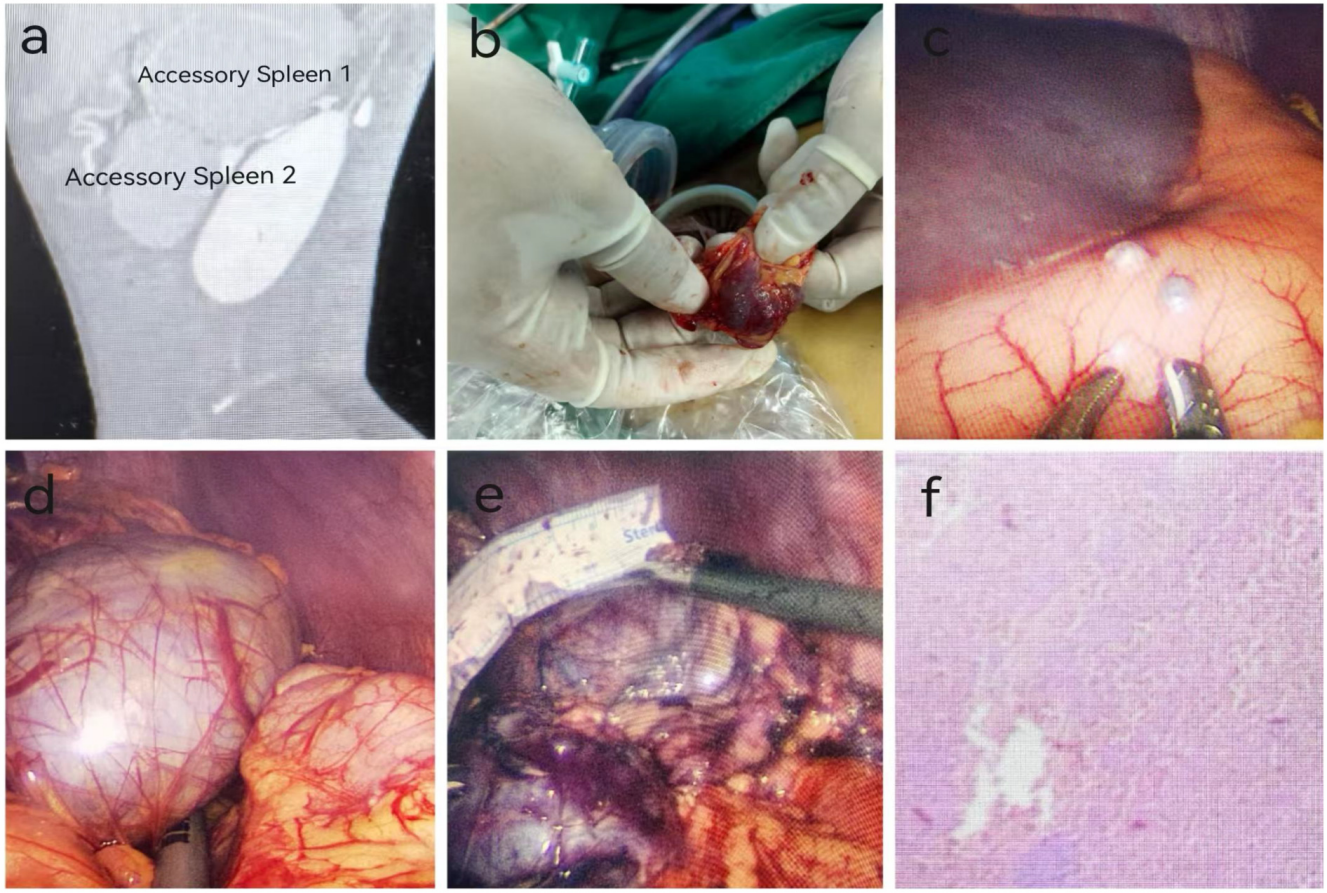
Preoperative examination, intraoperative findings, and postoperative pathology of a pediatric patient with accessory splenic hyperfunction treated via single-port plus one-port laparoscopic surgery (patient: male, 13 years old, diagnosed with accessory splenic hyperfunction). **(a)** Preoperative CT scan; **(b)** Accessory spleen implanted on the greater omentum; **(c)** Accessory spleen implanted on the stomach; **(d)** Accessory spleen 2; **(e)** Accessory spleen 1; **(f)** Postoperative pathology (Hematoxylin and Eosin staining, ×100 magnification). Pathological findings indicated atrophy and reduction of white pulp in the accessory spleen, with marked congestion in the red pulp, consistent with changes associated with accessory splenic hyperfunction.

A 13-year-old male child was admitted on July 23, 2024, at 16:12, presenting with jaundice for over a year. Medical history: 12 years ago, the child visited a local hospital for pale yellowish skin and was diagnosed with hemolytic anemia. He was later referred to Beijing Children's Hospital, where hemolysis tests indicated hereditary spherocytosis. In 2017, he underwent laparoscopic splenectomy at another hospital, during which two accessory spleens were observed, measuring approximately 2.0 cm*1.5 cm and 1.5 cm*1.5 cm, respectively, and were preserved. The child had been followed up regularly thereafter. One year ago, the child again developed pale yellowish skin, and ultrasound suggested an enlarged accessory spleen. The outpatient diagnosis was: (1) enlarged accessory spleen, (2) hemolytic jaundice, (3) hereditary spherocytosis, (4) post-splenectomy. During the course, the child had a normal appetite, with no fever, vomiting, or abdominal bloating. There was mild jaundice of the skin and mucous membranes, and normal bowel movements, with no obvious abnormalities in appearance. Physical examination: The child was alert, with mild yellowing of the skin and sclerae. No abnormalities in the heart and lungs. The abdomen was slightly distended, with no tenderness. There was a palpable fullness in the left upper abdomen, and the liver and spleen were not palpable below the costal margin. Auxiliary examinations: On July 24, 2024, ultrasound at our hospital revealed two hypoechoic masses in the splenic region (suggestive of enlarged accessory spleens) measuring 7.5 cm*6.3 cm*5.9 cm and 4.8 cm*3.9 cm*3.8 cm. A contrast-enhanced abdominal CT on the same day showed two accessory spleens in the left upper abdomen, with arcuate compression changes in the splenic vein (Figure 1a). Based on the medical history and auxiliary examination findings, the diagnoses were: (1) hyperfunction of the accessory spleen, (2) hemolytic jaundice, (3) hereditary spherocytosis, and (4) post-splenectomy.

### 2.2 Surgical method

Under general anesthesia, a single-port plus one laparoscopic accessory spleen removal was performed. Two accessory spleens were excised, and five spleens were implanted. During the single-port laparoscopic procedure, three implanted spleens were observed on the omentum, with the largest measuring 1.5*1.0*1.0 cm³ (Figure 1b). Two implanted spleens were observed in the gastric body, with the largest measuring approximately 0.5*0.5*0.5 cm³ (Figure 1c). In the splenic region, two large accessory spleens were identified, measuring approximately 7.0*6.5*5.0 cm³ and 6.0*5.0*5.0 cm³. Using an ultrasonic scalpel, three implanted spleens in the omentum were electrocoagulated and excised with forceps. Two implanted spleens in the gastric body were excised with an ultrasonic scalpel. The gastric body was sutured with 4-0 absorbable sutures after excision. The two large accessory spleens in the splenic bed were difficult to remove due to their size and their firm adhesions to the gastric body, tail of the pancreas, and diaphragm. The omentum was mobilized, and a 2-0 suture was used to secure the omentum to the greater curvature of the stomach, facilitating exposure. Electrocoagulation was used to dissect the omentum and expose the splenic hilum. Rich vascularization of the accessory spleens was noted. The first accessory spleen, measuring approximately 6.0*5.0*5.0 cm³, was separated using ultrasonic coagulation (Figure 1d). An ultrasonic scalpel was used to electrocoagulate the second accessory spleen near the hilum, focusing on the surrounding nourishing vessels. Severe bleeding occurred near the tail of the pancreas, which hindered the visual field. A 5 mm Trocar was placed in the left abdomen, and a suction device was used to aspirate the blood and improve exposure. After achieving hemostasis with the ultrasonic scalpel, the accessory spleen was carefully dissected from the surrounding tissues, including the stomach, tail of the pancreas, and diaphragm. The accessory spleen was excised, measuring approximately 7.5*6.5*5.0 cm³ (Figure 1e). The splenic bed was irrigated with saline, confirming no active bleeding. A specimen retrieval bag was used to capture the two large accessory spleens, the bag was tightened, and the spleens were progressively broken up and removed.

## 3 Results

The patient successfully underwent a single-port plus one laparoscopic splenectomy. The total surgery time was 195 min, with an intraoperative blood loss of 600 ml. The slightly increased blood loss was attributed to the rich vascular supply of the accessory spleen and the tight adhesions between the accessory spleen and surrounding tissues after splenectomy. On the second postoperative day, the patient passed gas and was started on a liquid diet. Postoperative pathology showed atrophy and reduction of the white pulp in the accessory spleen, with prominent congestion of the red pulp, consistent with the changes seen in hyperfunction of the accessory spleen (Figure 1f). On the first postoperative day, the jaundice significantly improved, and the bilirubin levels rapidly decreased to normal (Table 1). There was also a rapid increase in both white blood cells and platelets (Table 2). The white blood cell count quickly rose on the first postoperative day and gradually decreased thereafter. This was mainly because the large accessory spleen had taken over the function of the spleen, which is an important immune organ and plays a role in regulating white blood cells. After the excision of the remaining accessory spleen, this regulatory mechanism was lost, leading to an increase in white blood cell count. Additionally, the postoperative stress response also contributed to a reactive rise in white blood cells, which gradually returned to normal. The increase in platelet count was primarily due to the spleen being the main site of platelet destruction. After splenectomy, platelet destruction was reduced, and the bone marrow, no longer under the regulatory influence of the spleen, produced more platelets. The decrease in hemoglobin on the first postoperative day was due to intraoperative blood loss, but it gradually increased afterward, confirming the manifestations of accessory spleen hyperfunction. Postoperatively, the patient was treated symptomatically with antibiotics and platelet-lowering therapy. The patient was discharged on the eighth day after surgery. A six-month follow-up showed no complications such as bleeding, wound infection, thrombosis, or adhesive intestinal obstruction.

**Table 1:** Pre- and postoperative changes in bilirubin.

Indicator	Preoperative	Postoperative day 1	Postoperative day 5	Postoperative day 8
Total Bilirubin (TBIL)	101.8 umol/L	53.7 umol/L	36.1 umol/L	17.9 umol/L
Direct Bilirubin (DBIL)	20.3 umol/Lg/L	17 umol/L	15.9 umol/L	8.4 umol/L
Indirect Bilirubin (IBIL)	81.5 umol/L	36.7 umol/L	20.2 umol/L	9.5 umol/L

**Table 2:** Pre- and postoperative changes in complete blood count (CBC).

Indicator	Preoperative	Postoperative day 1	Postoperative day 5	Postoperative day 8
White blood cell (WBC)	7.33*10^9 ^/L	29.37*10^9 ^/L	13.57*10^9 ^/L	6.85*10^9 ^/L
Hemoglobin (HB)	152 g/L	107 g/L	110 g/L	114 g/L
Platelet (PLT)	441*10^9 ^/L	388*10^9 ^/L	606*10^9 ^/L	914*10^9 ^/L
C-reactive Protein (CRP)	<0.5 mg/L	19.31 mg/L	21.22 mg/L	3.90 mg/L

## 4 Discussion

Hereditary spherocytosis (HS) is a common hereditary hematological disorder in children, characterized by spherocytes that are prone to hemolysis due to defects in the red blood cell (RBC) membrane (2). The defect in HS results in a decreased ability of spherocytes to deform as they pass through the splenic sinusoids, leading to their destruction and causing symptoms such as anemia, jaundice, and splenomegaly in affected children.Surgical splenectomy is the most effective treatment method, as it effectively eliminates the hemolytic process, restoring the levels of hemoglobin, reticulocytes, and bilirubin to normal (3). With the advancement of technology, laparoscopic surgical techniques have become highly refined.Laparoscopic splenectomy (LS) has become the standard procedure, while single-incision laparoscopic splenectomy (SILS) is considered the ideal approach, offering a flawless, scar-free cosmetic outcome (4).

The texture and function of the accessory spleen are similar to those of the spleen, and it also plays a role in the destruction of abnormal red blood cells. The treatment guidelines for HS recommend that accessory spleens be identified and removed during total splenectomy to reduce the likelihood of symptom recurrence (5). However, some scholars believe that the spleen is one of the most important peripheral immune organs in the body, with functions such as the production of immunoglobulins, regulation of circulating blood volume, removal of aged or dying cells, and clearance of pathogens from the bloodstream (6). With the increasing understanding of the complications, such as infections and thrombosis, that can occur after total splenectomy, as well as the growing recognition of the spleen's importance as an immune organ—especially in pediatric HS—partial splenectomy or the retention of accessory spleens is gradually being developed. Regarding the timing of splenectomy for children with HS, most researchers recommend delaying the procedure in infants and young children with severe HS until they are at least 5 years old, as the risk of infection in children under 5 can increase by 1%–5% (7). Some scholars propose that for children under 6 years old requiring splenectomy for severe HS, partial splenectomy may be considered (8). However, few studies, both domestically and internationally, have analyzed the clinical characteristics of accessory spleen remnants or partial splenectomy in HS patients. Research on accessory spleen remnants consists mostly of a few case reports (9), with limited follow-up time. This study analyzes the clinical data of a pediatric HS patient at Anhui Provincial Children's Hospital, who developed accessory spleen hyperfunction after splenectomy. It summarizes the clinical characteristics and potential risks of accessory spleen remnants or partial splenectomy, providing evidence to further improve the diagnostic and therapeutic protocols for HS.

This is a rare case report. The child underwent laparoscopic splenectomy for HS 7 years ago, during which two accessory spleens were identified and preserved. The reasons for the residual accessory spleen after the first surgery may include: (1) the accessory spleens were preserved during the initial procedure, effectively acting as partial splenectomy; (2) the accessory spleen located at the splenic hilum was difficult to expose, and its removal could easily lead to inadvertent intraoperative bleeding. In HS patients with residual accessory spleens after splenectomy, the persistent primary disease causes continuous “stimulation” of hypertrophy in the remaining spleen or accessory spleen. Although initially small in size and often asymptomatic, this may eventually lead to the recurrence of hemolytic anemia and jaundice symptoms (10). In this case, during the regular follow-up after the first surgery, the residual accessory spleen gradually enlarged within 3 years postoperatively, and bilirubin levels continued to rise. However, the clinical parameters remained stable between 3 and 5 years post-surgery. After 6 years, the child developed significant jaundice and fatigue, which severely affected the quality of life. The accessory spleen grew rapidly, reaching a size equivalent to two adult spleens, and hyperfunction of the accessory spleen occurred. Over the past year, there was a significant increase in bilirubin levels: total bilirubin >100 µmol/L, direct bilirubin >20 µmol/L, and indirect bilirubin >70 µmol/L. In July 2024, the child underwent a second surgical intervention at our hospital, resulting in significant improvement in jaundice postoperatively.Preoperative total bilirubin was 101.8 µmol/L. On the first postoperative day, it decreased to 53.7 µmol/L, on the fifth day to 36.1 µmol/L, and on the eighth day to 17.9 µmol/L. Preoperative direct bilirubin was 20.3 µmol/L. On the first postoperative day, it decreased to 17 µmol/L, on the fifth day to 15.9 µmol/L, and on the eighth day to 8.4 µmol/L. Preoperative indirect bilirubin was 81.5 µmol/L. On the first postoperative day, it decreased to 36.7 µmol/L, on the fifth day to 20.2 µmol/L, and on the eighth day to 9.5 µmol/L. Bilirubin levels rapidly returned to normal, and hemolysis improved significantly. Chronic hemolysis increases the destruction of red blood cells, leading to an increase in unconjugated bilirubin, which causes liver metabolic disturbances and promotes the formation of pigment gallstones. Ali et al. conducted a retrospective study on 65 children with hereditary spherocytosis, finding that 20 of them had cholelithiasis, of which 13 were diagnosed before surgery. Studies on the incidence of cholelithiasis after splenectomy show that in a group of 130 patients who underwent partial splenectomy, 15 developed gallstones, while only 2 out of 182 patients who underwent total splenectomy developed gallstones (11). As for the risk of infections after splenectomy in younger children, recent years have seen preoperative vaccination against certain bacterial pathogens, such as Haemophilus influenzae type b, Streptococcus pneumoniae, and Neisseria meningitidis, as well as postoperative penicillin prophylaxis and early antibiotic treatment, which have significantly reduced the incidence of life-threatening infections (12, 13). A study by Luoto reported that among 49 children with hereditary spherocytosis who were vaccinated before splenectomy, the severe infection rate was 0% (14). Taha (15) reported that laparoscopic splenectomy is a safe and effective treatment for 71 children under the age of five with sickle cell disease. None of the patients developed fulminant infections when they received preoperative vaccinations and penicillin prophylaxis. The study also indicated that when there are indications for splenectomy, it is not necessary to wait until the patient reaches five years of age to perform the procedure. Preservation of splenic function means a risk of hemolysis and subsequent transfusion therapy or a need for secondary total splenectomy due to anemia. Pincez et al. reported on 79 children with hereditary spherocytosis who underwent partial splenectomy before the age of 5, most of whom were dependent on transfusions (31/39). After partial splenectomy (with a mean follow-up of 12 ± 0.9 years), the transfusion rate decreased, and hemoglobin levels increased. However, due to continued hemolysis, 50% of the 39 severe cases of hereditary spherocytosis underwent total splenectomy within 5 years after partial splenectomy (16). Gerhard et al. conducted a prospective study on 30 children with hereditary spherocytosis who underwent near-total splenectomy, with follow-up periods of 3–6 years. The average Hb increased by 2.9–5.0 g/dl, and bilirubin decreased by 15.4–56.4 μmol/L. These findings showed a significant reduction in hemolysis recurrence, spleen regeneration, and the need for subsequent surgeries (17). After splenectomy, the risk of arterial and venous thrombosis increases, typically including early acute splenic and portal vein thrombosis (SPVT) or later cardiovascular events, such as pulmonary hypertension. Acute SPVT is a life-threatening early complication after splenectomy, which can lead to intestinal ischemia or portal hypertension. Krauth et al. published a prospective and retrospective study, finding that 11 out of 89 patients who underwent splenectomy for hemolytic diseases developed SPVT (12.3%). In contrast, none of the 122 patients who underwent splenectomy due to splenic trauma developed SPVT (18, 19). The surgery itself did not appear to affect the incidence of SPVT but was rather associated with the underlying disease. One study (20) indicated that HS children who underwent partial splenectomy had a lower rate of platelet increase compared to those who underwent total splenectomy, which may reduce the postoperative thrombotic risk. Another study involving 26 children with hereditary spherocytosis recorded hemoglobin and platelet counts before and after total splenectomy, with follow-up periods ranging from 4 months to 19 years. No vascular events were observed in the children, possibly due to better vascular elasticity in children and the ability to stabilize platelet counts within normal ranges through medication postoperatively (21).

Splenectomy is a highly beneficial surgical strategy for treating children with moderate to severe hereditary spherocytosis, as it can reduce the need for blood transfusions and the occurrence of hyperbilirubinemia, with significant improvements observed after total splenectomy. Compared to children who undergo total splenectomy, those who undergo partial splenectomy have a higher incidence of cholelithiasis. Additionally, due to the recurrence of hemolysis after partial splenectomy, there is a risk of requiring a second surgery for total splenectomy. Reports of diseases caused by accessory spleens following splenectomy are relatively rare. Yao et al. (22) described a case of splenic hyperfunction in a patient after splenectomy, complicated by partial colonic obstruction caused by compression from a large accessory spleen. They also mentioned that in patients with hematological disorders requiring splenectomy, recurrence of the primary disease after splenectomy may occur, and laparoscopic examination revealed that accessory splenic hyperfunction was the cause. Removal of the accessory spleen resulted in a cure. Vladimir et al. (23) reported a case of laparoscopic removal of an occult accessory spleen and reviewed the literature, emphasizing the importance of preoperative diagnosis, intraoperative detection, and surgical removal of accessory spleens in patients with hematological diseases requiring splenectomy. Without these steps, accessory spleens could grow and lead to recurrence of the hematological disorder for which splenectomy was initially performed (24). Abdullah et al. reported a case of a 33-year-old male patient who experienced a recurrence of immune thrombocytopenic purpura 6 years after splenectomy, caused by hyperfunction of an intra-pancreatic accessory spleen (25). A study by Szold et al. also reported that 8 patients with recurrent immune thrombocytopenic purpura following splenectomy experienced symptom relief after the removal of the hyperfunctioning accessory spleen (26).

Due to the limited number of cases, it is currently not possible to quantify when clinical manifestations will appear. This child underwent a second surgery after 7 years of follow-up with a retained accessory spleen. Although this is an individual case, it provides valuable reference for the surgical management of HS in children, particularly those under 6 years old, when deciding whether to preserve the spleen or retain the accessory spleen. Our recommendation is that when children with hematological disorders require splenectomy, the procedure should be delayed until the child is over two years old, and performed after vaccination. We advise against preserving the accessory spleen or performing a partial splenectomy.

## Data Availability

The original contributions presented in the study are included in the article/Supplementary Material, further inquiries can be directed to the corresponding author.
